# An Overview of Listeriosis Outbreak Investigations in the United States Linked to Imported Enoki Mushrooms and Associated Regulatory Activities, Research, and Food Safety Knowledge Gaps

**DOI:** 10.1111/1541-4337.70292

**Published:** 2025-09-16

**Authors:** Stelios Viazis, Amanda Conrad, Alexandra Palacios, Alexandra L. Busbee, Margaret Kirchner, Natalie Cataldo, Allison Wellman, Brett Weed, Christina K. Carstens, Victoria Wagoner, Laurel S. Burall, Joelle K. Salazar, Megan L. Fay, Kenneth Nieves, Zachary Miller, David T. Ingram, Stephen Hughes, Joseph Baugher, Robert Hatch, Scott Izyk, Grace Pederson, Allison Scott, Matthew C Kristof, Laurie Williams, Lauren Yeung, Cary Chen Parker, Michael C. Bazaco

**Affiliations:** ^1^ Human Foods Program Food and Drug Administration College Park Maryland USA; ^2^ Centers for Disease Control and Prevention Atlanta Georgia USA; ^3^ Oak Ridge Institute for Science and Education Oak Ridge Tennessee USA; ^4^ Human Foods Program Food and Drug Administration Bedford Park Illinois USA; ^5^ Office of Inspections and Investigations Food and Drug Administration Rockville Maryland USA

**Keywords:** enoki mushrooms, *Listeria monocytogenes*, outbreaks

## Abstract

Foods implicated in listeriosis outbreaks continue to change over time. Historically, listeriosis outbreaks have been primarily linked to consumption of deli meat and dairy products. More recently, they have been linked to vegetable row crops, fruits, and other produce, including imported specialty mushrooms. Specialty mushrooms, including enoki mushrooms, are popular in Japanese, Chinese, and Korean cuisines, and are commonly used in soups, stir‐fries, hotpots, and salads. These mushrooms are imported into the U.S. from a variety of East Asian countries and Canada. Recently, food safety authorities around the world have linked listeriosis outbreaks to contaminated enoki mushrooms, leading to a series of highly impactful recalls. This review examines outbreaks and recalls associated with enoki mushrooms, related risks and food safety concerns, ongoing research, regulatory activities focused on this commodity, consumer preparation and handling practices, and prevention strategies. The exchange of epidemiologic and traceback evidence, genomic data, and international data sharing helped investigators find the source of multiple listeriosis outbreaks linked to enoki mushrooms. Since enoki mushrooms were first linked to listeriosis illnesses in 2020, state, federal, and international partners developed a strategy for outbreak prevention, including enhanced surveillance and an improved investigational approach for this commodity.

## 1 Introduction

Invasive listeriosis is a high‐priority foodborne illness due to its severity and disproportionate impact on high‐risk populations, such as newborns, pregnant women, and elderly people, as well as people with compromised immune systems (Pohl et al. 2019). In the U.S., approximately 1250 cases of listeriosis occur each year, resulting in 1070 hospitalizations and 172 deaths (Scallan et al. 2019). The pathogen that causes listeriosis, *Listeria monocytogenes*, is a challenge to control in the food industry as it is found ubiquitously in the food production chain and is psychrotolerant and halotolerant, making typical control measures less effective (Lee et al. 2013; Fotopoulou et al. 2024; Liu et al. 2005; Miller et al. 1997).

Foods frequently associated with listeriosis outbreaks have changed over time. While past listeriosis outbreaks were mostly linked to deli meats and soft cheeses, recent outbreaks have been linked to a variety of other foods, including packaged salads (U.S. Centers for Disease Control and Prevention 2022b) and frozen vegetables (Madad et al. 2023). Current attribution estimates of listeriosis across food categories show a broad distribution of foods, with the highest percentages linked to dairy (37.3%), vegetable row crops (22.6%), fruit (17.3%), and other produce (9.0%), which includes mushrooms (Interagency Food Safety Analytics Collaboration 2024).

In the United States, *L. monocytogenes* clinical isolates are whole genome sequenced (WGS) by state laboratories and uploaded to the public U.S. National Center for Biotechnology Information (NCBI) database and PulseNet USA. PulseNet USA identifies listeriosis clusters with three or more clinical isolates that are genetically related by ≤ 25 allele differences using whole genome multilocus sequence typing (wgMLST), uploaded within a 120‐day period (Tolar et al. 2019; U.S. Centers for Disease Control and Prevention 2016). The *Listeria* Initiative (LI) is a national surveillance system managed by the CDC that collects comprehensive clinical and epidemiologic data of all ill people with invasive listeriosis. State and local health departments attempt to interview all ill people, or their surrogates, with the standard LI case report form (U.S. Centers for Disease Control and Prevention 2024). The form collects demographic information, illness outcomes, and detailed food histories. When needed, ill people are re‐interviewed with a supplemental questionnaire by state and local health officials to obtain additional details on exposure information.

Identifying and investigating listeriosis outbreaks can be challenging, due to the nature of *L. monocytogenes* and its ability to survive in an environment for long periods. Contamination of a food product may occur at low levels, resulting in small numbers of illnesses over multiple years. Foodborne illness clusters are detected using certain thresholds, such as a certain number of cases within a time period, to identify potential outbreaks warranting an investigation. The pathogen's high morbidity and mortality and long incubation period are significant hurdles for gathering sufficient food exposure data to identify a suspected food vehicle, particularly when there are only a limited number of illnesses over many years. The application of WGS for all *L. monocytogenes* clinical isolates in the United States, starting in 2013, helped identify and solve long‐term outbreaks (Jackson et al. 2016). WGS has also enabled investigators to conduct sample‐initiated retrospective outbreak investigations (SIROI) by linking food and environmental isolates to genomically similar clusters of clinical isolates. The SIROI process has allowed for earlier outbreak detection with fewer illnesses, and earlier hypothesis generation and identification of a possible food vehicle for numerous *Listeria* outbreak investigations (Wellman et al. 2023).

The volume of food imported into the United States has steadily increased in the past 15 years. As of 2019, 32% of fresh vegetables and 55% of fresh fruit were supplied by other countries (U.S. Food and Drug Administration 2019a). The United States imports food from over 200 countries or territories with approximately 125,000 exporting food facilities and farms supplying the domestic market (U.S. Food and Drug Administration 2019a). The volume of food imported into the United States contributes to the complexity of the food supply but is also a prominent reason why the Food and Drug Administration's (FDA) food safety regulations extend beyond US borders. One example of a specialty mushroom that is imported into the United States is the enoki mushroom, also known as an enokitake, golden needle, futu, seafood, or lily mushroom (U.S. Food and Drug Administration 2022). Popular in East Asian cuisines, these long, thin, white mushrooms are characterized by a mild flavor and crunchy texture and are commonly used in soups, stir‐fries, hotpots, and salads. Enoki mushrooms are imported into the United States from Japan, the People's Republic of China*, the Republic of Korea*, Taiwan, and Canada (U.S. Food and Drug Administration 2022) (*hereafter referred to as China and Korea, respectively).

*L. monocytogenes* has been isolated from samples of various mushrooms in both North America and China (Chen et al. 2018). In 2019, a potential contamination link between *L. monocytogenes* and enoki mushrooms was identified following regulatory sampling that subsequently led to a recall in Canada (Government of Canada 2019). In 2020, the first outbreak of *L. monocytogenes* infections, identified through an SIROI, was linked to enoki mushrooms imported from a manufacturer in Korea, leading to a total of 48 ill people: 36 in the United States and 12 in Canada (Pereira et al. 2023). Concurrent to the investigation, health officials in Australia investigated six listeriosis illnesses with clinical isolates related to the outbreak strain with illness onset dates between 2017 and 2020 (Food Standards Australia and New Zealand 2023). Prior to this outbreak, federal and state investigative partners were not aware of outbreaks linked to imported enoki mushrooms reported in the previous 20 years, although these are extremely challenging to discern. Without samples yielding the outbreak strain, identifying enoki mushrooms as a potential vehicle has been nearly impossible from epidemiologic data alone. Prior to 2016, fresh mushrooms were not included on standardized questionnaires, and enoki mushrooms specifically were not included; multiple re‐interviews are often required, and patients may not recall enoki ingredients in a meal. In 2022, the US and Canadian authorities conducted a second binational SIROI of *L. monocytogenes* infections linked to enoki mushrooms imported from China, resulting in six illnesses. These outbreaks highlight the importance of surveillance testing and collaboration between partners to identify and stop foodborne outbreaks on a global scale (Kirchner et al. 2025).

Public health and regulatory agencies in the U.S. and Canada are committed to conducting comprehensive surveillance for *Listeria* in foods and in people, efficiently investigating identified outbreaks, and implementing control measures to potentially minimize the impact of future outbreaks. Here, we examine current knowledge regarding associated listeriosis outbreaks, commodity risks and food safety concerns, ongoing research on *L. monocytogenes* and enoki mushrooms, and regulatory activities focused on enoki mushrooms.

## 2 Enoki Mushrooms

### 2.1 Outbreak

The first identified listeriosis outbreak definitively linked to enoki mushrooms from Korea included illnesses from the United States and Canada (Pereira et al. 2023). In February 2020, the Canadian Food Inspection Agency (CFIA) uploaded two mushroom samples to NCBI that were highly related to isolates from an outbreak in the United States that the CDC had opened investigations for three times (in 2017, 2018, and finally in 2020, as cases continued to occur). The samples were from a sliced portobello mushroom collected in 2016 and an enoki mushroom sample collected in 2019. The Canadian sample served as the catalyst for the outbreak investigation since it prompted US investigators to ask ill people specifically about mushroom exposure, including enokis. As a result, Michigan state partners targeted enokis for sampling, after an ill person reported purchasing them, and investigators recovered the outbreak strain. The mushrooms were ultimately identified as the vehicle causing the illnesses, and a recall was initiated. In total, the US outbreak included 36 ill people from 17 states with specimen collection dates ranging from November 23, 2016, to December 13, 2019. All isolates were related within 0–38 allele differences by wgMLST.

The FDA performed a WGS analysis using the CFSAN SNP Pipeline, a reference‐based variant detection method, which found that 67 human and food isolates associated with this outbreak were within 23 SNPs of each other and determined to be a match (Davis et al. 2015). Of 28 ill people for whom data were available, ages ranged from <1 to 96 years, with a median age of 67; 42% were male, and 17 (61%) reported Asian ancestry. Six illnesses were associated with pregnancy; two resulted in fetal loss. Of the 33 ill people with outcome information, 31 were hospitalized (94%), and there were four deaths. Exposure information was available for 22 ill people; 12 (55%) reported consuming mushrooms, with one ill person reporting enoki mushrooms. Canada identified 12 illnesses related to the outbreak strain with specimen collection dates ranging from August 4, 2016, to February 10, 2019. The investigation linked enoki mushrooms from a manufacturer in Korea as the source of the outbreak. Epidemiologic, laboratory, and traceback evidence led to multiple regulatory actions, including extensive voluntary recalls by three firms in the United States and one firm in Canada. The Korean manufacturer was also placed on an import alert in the United States and Canada.

### 2.2 Suspected Outbreak

In June of 2021, the CDC identified enoki mushrooms as the suspect vehicle for another listeriosis outbreak. Five ill people were included from five states with specimen collection dates ranging from September 13, 2017, to March 27, 2021. Ages ranged from 29 to 83 years, with a median age of 60, and 20% of ill people were male. Three ill people were identified as White (60%), and two were identified as Asian (40%). All five ill people were hospitalized, and there were no reported deaths. One ill person was pregnant and remained pregnant at the time of the interview. Of four people with food histories available, one ill person reported exposure to fresh mushrooms but did not specify the type. In addition to five clinical isolates, three enoki mushroom samples collected in May 2021 from Korea were included in this investigation. All eight isolates were related within 2–34 allele differences by wgMLST. The FDA analysis found that 14 isolates associated with this outbreak were within 17 SNPs of each other and determined to be a match, including clinical isolates from Australia and Korea and an Australian food isolate. CDC closed this outbreak investigation in July 2021 with enoki mushrooms identified as the suspect vehicle due to the genetic link between the clinical cluster and the enoki mushroom isolates. Epidemiologic information was limited, and the vehicle could not be confirmed.

### 2.3 Outbreak

In 2022, US and Canadian partners investigated a binational SIROI (Wellman et al. 2023) of *L. monocytogenes* infections linked to enoki mushrooms, which was the second outbreak of listeriosis with a confirmed link to enoki mushrooms (U.S. Centers for Disease Control and Prevention 2023b; Kirchner et al. 2025). The enoki mushrooms implicated in this outbreak were imported from China. This outbreak included two genetically unrelated strains of *L. monocytogenes* (outbreak strains one and two) that were investigated as a single outbreak, because both strains were found on a single sample of enoki mushrooms from the same firm in China. This outbreak included six ill people from four states and Canada. Specimen collection dates ranged from October 3, 2022, to February 3, 2023. Ages ranged from <1 to 71 years with a median age of 49, and 50% of ill people were male. Three ill people were identified as Asian, and two were identified as Hispanic. All six ill people were hospitalized, and there was one reported death (it was not determined if *Listeria* contributed to the death). There was one pregnancy‐associated illness in a newborn who survived; the mother was not considered a case in this outbreak. Two ill people reported consuming enoki mushrooms: One from a grocery store in a spring roll and the other at a restaurant in a hotpot dish. Four ill people denied enoki mushroom consumption; three reported shopping at East Asian‐style grocery stores or eating at East Asian‐style restaurants. The first outbreak strain was recovered from six enoki mushroom samples from China and was related to four clinical isolates within 0–29 allele differences by wgMLST. This strain was also isolated from ill people in China. The second outbreak strain was recovered from twelve enoki mushroom samples from China and was related to two clinical isolates within 0–44 allele differences by wgMLST. Overall, the two outbreak strains differed by over 1900 allele differences by wgMLST.

The FDA analysis found that Strain 1 included 12 isolates, which were within 29 SNPs of each other and determined to be a match. Strain 2 included 14 isolates, which were within 20 SNPs of each other and determined to be a match. Epidemiologic, laboratory, and traceback evidence led to voluntary recalls by firms and the issuance of a country‐wide import alert for China by the FDA. Retail product testing performed by states and investigations at firms in the United States resulted in several recalls, including two recalls of enoki mushrooms from retail samples found to have non‐outbreak strains of *L. monocytogenes*. The FDA and CDC issued outbreak advisories to the public and warned consumers not to eat recalled enoki mushrooms (U.S. Food and Drug Administration 2023d; U.S. Centers for Disease Control and Prevention 2022a). As a result of this outbreak, CDC continues to advise people at higher risk not to eat raw enoki mushrooms while CDC works to assess the risk of listeriosis from the consumption of enoki mushrooms.

### 2.4 Suspected Outbreak Retrospectively Identified, as Part of a 2021–2023 Investigation

In 2021, the CDC conducted a retrospective project to analyze clusters related to enoki mushroom isolates. In 2021, the FDA uploaded enoki mushroom isolates that were related to an outbreak investigated by the CDC in 2015. The outbreak included seven illnesses, and no suspected vehicle was identified. CDC assessed ancestry and mushroom exposure information of ill people to see if enoki mushrooms could be a plausible suspect vehicle for this outbreak. This outbreak included 14 ill people from eight states with specimen collection dates ranging from August 14, 2014, to August 10, 2023. Clinical isolates were genetically related to five enoki mushroom isolates that the FDA recovered from four samples collected in July and August 2021 from Korea. Outbreak isolates were also related to food and environmental isolates from the United Kingdom and Australia. All isolates were related within 0–38 allele differences by wgMLST. The FDA analysis found that 26 isolates associated with this outbreak were within 21 SNPs of each other and determined to be a match, including clinical isolates from Australia and Korea. Ages ranged from <1 to 91 years, with a median age of 57, and 21% of ill people were male. Ancestry information was available for ten ill people, and six reported Asian ancestry (60%). Outcome information was available for ten ill people; all were hospitalized, and there were two reported deaths. Three illnesses were associated with pregnancy, including two newborns. Mushroom exposure information was available for three people, and two reported consuming mushrooms, but not specifically enoki mushrooms. Prior to 2016, fresh mushrooms were not on the standard LI case report form, which resulted in many ill people who were not asked about fresh mushroom exposure. Retrospectively, enoki mushrooms were designated as the leading hypothesis of illnesses on the basis of epidemiologic and laboratory data, but could not be confirmed as the source.

### 2.5 *L. monocytogenes* Isolates in the NCBI Pathogen Detection Database Linked to Enoki Mushrooms

As of December 5, 2023, WGS data were available in the public NCBI Pathogen Detection database for 247 *L. monocytogenes* isolates from samples of enoki mushrooms collected by the FDA and nine state health agencies (Table 1). The sampled enoki mushrooms originated from China (110 isolates), Korea (103), Taiwan (29), and Japan (5). These isolates fall into 24 distinct NCBI SNP clusters (along with four isolates that are not part of a cluster), illustrating significant genetic diversity of these *L. monocytogenes* strains. Based on FDA analysis, these enoki mushroom isolates match clinical isolates from Australia, Cambodia, Canada, China, Germany, Korea, Taiwan, the United Kingdom, and the United States, highlighting a possible link to human illness across the globe. PulseNet identified ten clusters of *L. monocytogenes* that included both clinical and enoki mushroom isolates related within 25 alleles by wgMLST. Five clusters met PulseNet's *L. monocytogenes* cluster detection threshold and were investigated by CDC, as discussed above. The remaining five clusters included 16 clinical isolates and did not meet PulseNet's *L. monocytogenes* cluster detection threshold for investigation by CDC. The clusters included illnesses occurring over long periods of time, ranging from 2 to 16 years. CDC retrospectively reviewed LI forms for ill people and assessed ancestry, grocery stores, restaurants, and mushroom exposures to see if these clusters had any signals for enoki mushrooms or East Asian‐style foods. Although fresh mushrooms were added to the LI form in 2016, the question relies on the ill person to provide the types of mushrooms they consumed rather than asking about specific types, such as enoki. It is difficult to determine enoki mushroom exposure based on what is reported on the LI form, which limits our assessment of whether a person ate or likely ate enoki mushrooms. Based on available information, two clusters had epidemiologic data consistent with enoki mushrooms as a suspect vehicle.

**TABLE 1:** Public Actions and Notifications associated with enoki mushrooms, 2020–2024.

Public communication	Date	Sample that initiated the recall/advisory	Recall related to outbreak?
Sun Hong Foods, Inc. Recalls Enoki Mushroom Because of Possible Health Risk, FDA (https://www.fda.gov/safety/recalls‐market‐withdrawals‐safety‐alerts/sun‐hong‐foods‐inc‐recalls‐enoki‐mushroom‐because‐possible‐health‐risk)	3/9/2020	State sample, Michigan Department of Agriculture and Rural Development	Yes
Guan's Mushroom Co Recalls Enoki Because of Possible Health Risk, FDA (https://www.fda.gov/safety/recalls‐market‐withdrawals‐safety‐alerts/guans‐mushroom‐co‐recalls‐enoki‐because‐possible‐health‐risk)	3/23/2020	State sample, California Department of Public Health	Yes
H&C Food Inc. Recalls Enoki Mushroom Because of Possible Health Risk, FDA (https://www.fda.gov/safety/recalls‐market‐withdrawals‐safety‐alerts/hc‐food‐inc‐recalls‐enoki‐mushroom‐because‐possible‐health‐risk)	4/7/2020	FDA import sample confirmed foreign supplier as source of outbreak	Yes
Guan's Mushroom Co Recalls Enoki Because of Possible Health Risk, FDA (https://www.fda.gov/safety/recalls‐market‐withdrawals‐safety‐alerts/guans‐mushroom‐co‐recalls‐enoki‐because‐possible‐health‐risk‐0#:~:text=Guan%27s%20Mushroom%20Co%20of%20Commerce,frail%20or%20elderly%20people%2C%20and)	4/20/2021	State sample, Michigan Department of Agriculture and Rural Development	Yes
Golden Medal Mushroom Inc. Recalls Enoki Mushrooms Because of Possible Health Risk, FDA (https://www.fda.gov/safety/recalls‐market‐withdrawals‐safety‐alerts/golden‐medal‐mushroom‐inc‐recalls‐enoki‐mushrooms‐because‐possible‐health‐risk)	4/22/2021	State sample, Michigan Department of Agriculture and Rural Development	No
Revised Guan's Mushroom Co Recalls Enoki Because of Possible Health Risk, FDA (https://www.fda.gov/safety/recalls‐market‐withdrawals‐safety‐alerts/revised‐guans‐mushroom‐co‐recalls‐enoki‐because‐possible‐health‐risk)	5/6/2021	State sample, Massachusetts Department of Public Health	Yes
Sun Hong Foods, Inc. Recalls Seafood Mushroom Because of Possible Health Risk, FDA (https://www.fda.gov/safety/recalls‐market‐withdrawals‐safety‐alerts/sun‐hong‐foods‐inc‐recalls‐seafood‐mushroom‐because‐possible‐health‐risk)	5/28/2021	State sample, California Department of Public Health	No
California Terra Garden Inc. Recalls Seafood Mushroom Because of Possible Health Risk, FDA (https://www.fda.gov/safety/recalls‐market‐withdrawals‐safety‐alerts/california‐terra‐garden‐inc‐recalls‐seafood‐mushroom‐because‐possible‐health‐risk)	5/28/2021	State sample, California Department of Public Health	No
Rainfield Marketing Group, Inc. of Vernon, CA is Recalling Enoki Mushrooms (Product of Korea) Because it has the Potential to be Contaminated with Listeria monocytogenes, FDA (https://www.fda.gov/safety/recalls‐market‐withdrawals‐safety‐alerts/rainfield‐marketing‐group‐inc‐vernon‐ca‐recalling‐enoki‐mushrooms‐product‐korea‐because‐it‐has)	5/28/2021	State sample, California Department of Public Health	No
Concord Farms Recalls Enoki Mushrooms Due to Possible Health Risk, FDA (https://www.fda.gov/safety/recalls‐market‐withdrawals‐safety‐alerts/concord‐farms‐recalls‐enoki‐mushrooms‐due‐possible‐health‐risk)	5/28/2021	State sample, California Department of Public Health	No
Marquis Worldwide Specialty Inc. Recalls Organic Enoki Mushroom 200 g Because of Possible Health Risk, FDA (https://www.fda.gov/safety/recalls‐market‐withdrawals‐safety‐alerts/marquis‐worldwide‐specialty‐inc‐recalls‐organic‐enoki‐mushroom‐200g‐because‐possible‐health‐risk)	5/28/2021	State sample, California Department of Public Health	No
MDARD Issues Consumer Advisory for Certain Enoki Mushrooms	12/3/2021	LFFM sample. Michigan Department of Agriculture and Rural Development	No
Golden Medal Mushroom Inc. Recalls Enoki Mushrooms Because of Possible Health Risk, FDA (https://www.fda.gov/safety/recalls‐market‐withdrawals‐safety‐alerts/golden‐medal‐mushroom‐inc‐recalls‐enoki‐mushrooms‐because‐possible‐health‐risk‐0)	2/3/2022	LFFM sample, Michigan Department of Agriculture and Rural Development	No
Jan Fruits Inc. Recalls Enoki Mushrooms Because of Possible Health Risk, FDA (https://www.fda.gov/safety/recalls‐market‐withdrawals‐safety‐alerts/jan‐fruits‐inc‐recalls‐enoki‐mushrooms‐because‐possible‐health‐risk)	2/8/2022	LFFM sample, California Department of Public Health	No
Concord Farms Recalls Enoki Mushrooms Due to Possible Health Risk, FDA (https://www.fda.gov/safety/recalls‐market‐withdrawals‐safety‐alerts/concord‐farms‐recalls‐enoki‐mushrooms‐due‐possible‐health‐risk‐0)	2/9/2022	LFFM sample, California Department of Public Health	No
Golden Medal Mushroom Inc. Recalls Enoki Mushrooms Because of Possible Health Risk (Revised), FDA (https://www.fda.gov/safety/recalls‐market‐withdrawals‐safety‐alerts/golden‐medal‐mushroom‐inc‐recalls‐enoki‐mushrooms‐because‐possible‐health‐risk‐revised)	2/23/2022	Expanded recall due to investigation	No
Jan Fruits Inc. Recalls Enoki Mushrooms Because of Possible Health Risk, FDA (https://www.fda.gov/safety/recalls‐market‐withdrawals‐safety‐alerts/jan‐fruits‐inc‐recalls‐enoki‐mushrooms‐because‐possible‐health‐risk‐0)	3/18/2022	LFFM sample, California Department of Public Health	No
Top Quality Produce, Inc. Recalls Enoki Mushroom Because Of Possible Health Risk, FDA (https://www.fda.gov/safety/recalls‐market‐withdrawals‐safety‐alerts/top‐quality‐produce‐inc‐recalls‐enoki‐mushroom‐because‐possible‐health‐risk)	3/18/2022	LFFM sample, California Department of Public Health	No
T Fresh Company Recalls Enoki Mushrooms Because of Possible Health Risk, FDA (https://www.fda.gov/safety/recalls‐market‐withdrawals‐safety‐alerts/t‐fresh‐company‐recalls‐enoki‐mushrooms‐because‐possible‐health‐risk)	3/19/2022	LFFM sample, California Department of Public Health	No
WISETRADE CORPORATION RECALLS ENOKI MUSHROOMS BECAUSE OF POSSIBLE HEALTH RISK, FDA (https://www.fda.gov/safety/recalls‐market‐withdrawals‐safety‐alerts/wisetrade‐corporation‐recalls‐enoki‐mushrooms‐because‐possible‐health‐risk)	3/23/2022	LFFM sample, California Department of Public Health	No
State Health Officials Warns Consumers Not to Eat Specific Brands of Imported Enoki and Mixed Mushrooms Because They May Cause Illness (https://www.cdph.ca.gov/Programs/OPA/Pages/NR22‐063.aspx)	3/25/2022	Mix of state and LFFM samples (all recalled). California Department of Public Health	No
T Fresh Company of City of Industry, CA is Recalling its 7.5oz (200 g) Yes! Enoki Mushrooms Due to Possible Health Risk, FDA (https://www.fda.gov/safety/recalls‐market‐withdrawals‐safety‐alerts/t‐fresh‐company‐city‐industry‐ca‐recalling‐its‐75oz‐200g‐yes‐enoki‐mushrooms‐due‐possible‐health)	4/20/2022	LFFM sample, California Department of Public Health	No
Green Day Produce, Inc. Recalls Enoki Mushrooms Because of Possible Health Risk, FDA (https://www.fda.gov/safety/recalls‐market‐withdrawals‐safety‐alerts/green‐day‐produce‐inc‐recalls‐enoki‐mushrooms‐because‐possible‐health‐risk)	11/17/2022	State sample, Michigan Department of Agriculture and Rural Development	No
Utopia Foods Recalls “Enoki Mushrooms” Because of Possible Health Risk, FDA (https://www.fda.gov/safety/recalls‐market‐withdrawals‐safety‐alerts/utopia‐foods‐recalls‐enoki‐mushrooms‐because‐possible‐health‐risk)	12/13/2022	LFFM Sample, Missouri Department of Health and Senior Services	Yes
FDA Advises Restaurants and Retailers Not to Serve or Sell and Consumers Not to Eat Product Labeled as Sun Hong Foods, Inc. Enoki Mushrooms Sourced from China Due to Possible Listeria Contamination, FDA (https://www.fda.gov/food/alerts‐advisories‐safety‐information/fda‐advises‐restaurants‐and‐retailers‐not‐serve‐or‐sell‐and‐consumers‐not‐eat‐product‐labeled‐sun)	12/17/2022	LFFM Sample, Missouri Department of Health and Senior Services	Yes
Utopia Foods Expands Recall on “Enoki Mushrooms” Because of Possible Health Risk, FDA (https://www.fda.gov/safety/recalls‐market‐withdrawals‐safety‐alerts/utopia‐foods‐expands‐recall‐enoki‐mushrooms‐because‐possible‐health‐risk)	1/13/2023	Expanded recall due to outbreak investigation	Yes
Maryland Department of Health issues consumer advisory for Enoki Mushrooms (https://health.maryland.gov/newsroom/Pages/Maryland‐Department‐of‐Health‐issues‐consumer‐advisory‐for‐Enoki‐Mushrooms.aspx)	1/25/2023	LFFM sample, Maryland Department of Health	No
Xin Ao International Group Corp. Recalls “Sss Enoki Mushroom” & “K‐Fresh Mushroom” Because of Potential Health Risk, FDA (https://www.fda.gov/safety/recalls‐market‐withdrawals‐safety‐alerts/xin‐ao‐international‐group‐corp‐recalls‐sss‐enoki‐mushroom‐k‐fresh‐mushroom‐because‐potential‐health)	2/17/2023	LFFM sample, Maryland Department of Health	No
Jan Fruits Inc. Recalls Enoki Mushrooms Because of Possible Health Risk, FDA (https://www.fda.gov/safety/recalls‐market‐withdrawals‐safety‐alerts/jan‐fruits‐inc‐recalls‐enoki‐mushrooms‐because‐possible‐health‐risk‐1)	2/24/2023	LFFM sample, Hawaii Department of Health	No
News Releases from Department of Health: Recalled enoki mushrooms sold in Hawaiʻi associated with potential Listeria contamination (https://health.hawaii.gov/news/newsroom/recalled‐enoki‐mushrooms‐sold‐in‐hawaii‐associated‐with‐potential‐listeria‐contamination/)	2/27/2023	LFFM sample, Hawaii Department of Health	No
CONSUMER ALERT: Listeria Monocytogenes in Qilu Enterprises Brand “Enoki Mushrooms”; Agriculture and Markets (https://agriculture.ny.gov/news/consumer‐alert‐listeria‐monocytogenes‐qilu‐enterprises‐brand‐enoki‐mushrooms)	10/21/2023	LFFM sample, West Virginia Department of Health and Human Resources and West Virginia Department of Agriculture	No
Utopia Foods Recalls “Enoki Mushrooms” Because of Possible Health Risk, FDA (https://www.fda.gov/safety/recalls‐market‐withdrawals‐safety‐alerts/utopia‐foods‐recalls‐enoki‐mushrooms‐because‐possible‐health‐risk‐0?utm_medium=email&utm_source=govdelivery)	10/23/2023	LFFM sample, West Virginia Department of Health and Human Resources and West Virginia Department of Agriculture	No
Enoki King Mushroom Farm Recalls Enoki Because of Possible Health Risk, FDA (https://www.fda.gov/safety/recalls‐market‐withdrawals‐safety‐alerts/enoki‐king‐mushroom‐farm‐recalls‐enoki‐because‐possible‐health‐risk)	10/11/2024	LFFM sample, Maryland Department of Health	No
Maryland Department of Health issues consumer advisory for Enoki Mushrooms (https://health.maryland.gov/newsroom/Pages/Consumer‐advisory‐for‐Enoki‐Mushrooms.aspx)	10/17/2024	LFFM sample, Maryland Department of Health	No
HH Fresh Trading Corp Recalls Taiwan Enoki 200gx25pk Because of Possible Health Risk, FDA (https://www.fda.gov/safety/recalls‐market‐withdrawals‐safety‐alerts/hh‐fresh‐trading‐corp‐recalls‐taiwan‐enoki‐200gx25pk‐because‐possible‐health‐risk)	11/1/2024	LFFM sample, West Virginia Department of Health and Human Resources and West Virginia Department of Agriculture	No
New Age International Recalls ‘Enoki Mushrooms’ Due to Potential Health Risk, FDA (https://www.fda.gov/safety/recalls‐market‐withdrawals‐safety‐alerts/new‐age‐international‐recalls‐enoki‐mushrooms‐due‐potential‐health‐risk)	12/11/2024	LFFM sample, Maryland Department of Health	No

As of December 5, 2023, WGS data were available in the public NCBI Pathogen Detection database for 114 *Listeria innocua* isolates from samples of enoki mushrooms collected by the FDA and four state health agencies (Table 1). These isolates fall into 15 distinct NCBI clusters (along with 10 isolates which are not part of a cluster), again illustrating the significant genetic diversity of these *L. innocua* strains (Settanni and Corsetti 2007). Continued findings of *L. innocua* may indicate conditions in the facility that are supportive of *L. monocytogenes* survival and growth, and therefore require additional attention to cleaning and sanitation (Tortorello 2003).

### 2.6 Enoki Mushroom Market Dynamics

The U.S. Department of Agriculture (USDA) defines specialty mushrooms as any that are not from the genus *Agaricus* (Crosby W. 2019) (any type other than button, cremini, or portabella varieties). Specialty mushroom production in the United States began in the 1980s, with enokis among the first species commercially cultivated in the United States at a farm in Washington state. As of 2022, domestic production of specialty mushrooms (other than shiitake and oyster) totaled 10.2 million pounds worth $45 million. Some market analysts believe the overall mushroom market has the capacity to grow to $20 billion domestically as consumers seek products that serve as meat substitutes (Morgan 2023; U.S. Department of Agriculture National Agricultural Statistics Service 2022).

Overall, mushroom consumption increased in the United States for nearly 50 years (U.S. Department of Agriculture Economic Research Service 2023), with per capita annual consumption reaching approximately three pounds per person as of 2017 (U.S. Department of Agriculture Economic Research Service 2017). In 2022, the United States was the world's top importer of fresh and refrigerated mushrooms, accounting for 91,000 metric tons of product valued at over $454 million (World Integrated Trade Solution 2022). While the enoki supply in the United States is primarily sourced from abroad, some domestic production does occur (U.S. Food and Drug Administration 2024a). Countries and regions exporting to the United States include known enoki producers such as Canada (#1; $333 million), China (#4; $13 million), Korea (#5; $12 million), Taiwan^1^ (#10; $0.8 million), and Japan (#12; $0.6 million).

Imports of specialty mushrooms into the United States increased in recent years, more than doubling since 2016 and increasing 226% from a decade ago (Table 2). Even as the import market grows rapidly, total domestic mushroom production, dominated by *Agaricus* (button and portabella) mushrooms, vastly outpaces imports. For the growing season ending in 2022, US domestic mushroom production reached 702 million pounds valued at just over $1 billion (U.S. Department of Agriculture National Agricultural Statistics Service 2022).

**TABLE 2:** United States imports of fresh and refrigerated mushrooms, 1992; 2002; 2012–2022 (World Integrated Trade Solution 2022).

Year	Value (millions)	Volume (metric tons)
2022	$454	91,472
2021	$410	88,673
2020	$349	81,167
2019	$319	76,485
2018	$277	68,311
2017	$231	60,460
2016	$208	56,122
2015	$186	50,724
2014	$160	45,651
2013	$144	44,771
2012	$139	43,341
2002	$59	26,561
1992	$4	2,053

(World Integrated Trade Solution 2022).

### 2.7 Taxonomy and Ecology of Enoki Mushrooms

Enoki mushrooms include two species within the *Flammulina* genus. This genus is typically associated with enoki mushrooms from the northern hemisphere, though enoki mushrooms from this genus have also been identified in the southern hemisphere, raising questions of their origin (Methven et al. 2000). Even before the identification of two species of enoki mushrooms, a study noted separation of *Flammulina velutipes* into three geographical groups (Methven et al. 2000). Recent data suggests speciation based on region and varieties from Asia may be from a new species, *Flammulina filiformis*, which was originally postulated to be a variant within *F. velutipes* (Wang et al. 2018; Ge et al. 2015). This determination is made based on the genetic sequence of five genes in the organisms. While morphological differences may be noted, they are not distinct enough to be a reliable determinant of speciation. Several names for mushrooms are derived from these species, including enoki/enokitake, winter mushrooms, velvet shank, golden needle mushrooms, velvet foot, futu, lily mushrooms, and seafood mushrooms (Dong et al. 2021). Given the limited genetic analysis and the breadth of names, enoki will be used as an aggregate to refer to all of these.

Enoki mushrooms grow in the wild, with varieties found in North America, Europe, Australia, and Asia. These mushrooms are found growing on hardwood, such as aspens, willows, and elms. The enoki gains one of its names, winter mushrooms, because it typically fruits in the late fall until early spring, as long as temperatures stay above freezing (Tonomura 1978). Primordia, which form the base for the fruiting bodies, form between 5°C and 20°C, though fruiting is less efficient at the outer edges of that range (Tonomura 1978). Most enoki mushrooms on sale are commercially cultivated instead of wild mushrooms (Gherman and Moran 2024). However, it is not known if wild enoki mushrooms present a similar *L. monocytogenes* risk compared to cultivated mushrooms (Gherman and Moran 2024).

### 2.8 Enoki Mushroom Growing and Packaging Practices

While there are no estimates on the number of home growers in the United States, the availability of enoki cultivation products in the United States and resources on the internet support this potential group of consumers and their use of growth practices similar to wild growth conditions. Growth substrates use an aged hardwood sawdust mixture, supplemented with a variety of options such as rice, wheat bran, or soybean hulls (Tonomura 1978; Sharma et al. 2021). The mixture is used to fill bags or jars, which are then sealed and sterilized. Prior to inoculation with the spawn, the jars are cooled to the initial incubation temperature (Sharma et al. 2021). Incubation temperatures are initially set for optimal mycelium infiltration of the substrate, which generally occurs at 22°C–25°C (Sharma et al. 2021). After infiltration, the surface of the substrate is scraped to clear space for primordia formation, and the cultures are transferred to a growing room that is 10°C–14°C to induce fruiting body formation, also referred to as pinning. Humidity in these rooms is increased to 80%–85% (Sharma et al. 2021). After pinning, cultures are scrolled or collared with plastic or paper sheaths to support stipe elongation.

Cultures are then transferred to a room with a lower temperature (8°C–12°C), and humidity may stay the same or be increased (Sharma et al. 2021). Increasing CO_2_ can also aid mushroom elongation. Engagement with competent authorities, mushroom producers, and data collected during inspections suggests that enoki mushrooms are harvested by hand, trimmed with a knife, weighed, and then vacuumed‐sealed, often in plastic packages, with no modified atmosphere and typically stored/transported under refrigerated conditions (less than 41°F) to maintain organoleptic quality. Specific conditions and practices vary depending on the grower and their choices of enoki variety and growing media. Fresh mushrooms have a short shelf‐life (3–14 days), depending on storage conditions. They can be dehydrated to extend this shelf life (6–12 months).

### 2.9 Contamination of Enoki Mushrooms

Little is known about the sources of *L. monocytogenes* contamination in enoki mushrooms. In addition, limited data about the survival and proliferation of *L. monocytogenes* during enoki mushroom cultivation are available. However, research demonstrates recurring prevalence of *L. monocytogenes* in enoki mushrooms. In 2014, researchers examined four enoki mushroom producers in China and collected samples throughout the cultivation process. The researchers detected *L. monocytogenes* at multiple stages in the production process and in 65% of the finished product samples (Chen et al. 2018). Results suggested the mycelium scraping machinery may serve as the primary contamination source of *L. monocytogenes* (Chen et al. 2018). Researchers subsequently collected enoki mushroom samples in 2015 and 2018. They found 76.3% (29/38) of enoki samples were positive for *L. monocytogenes* in 2015 (Chen et al. 2018), and 55.5% (116/209) of enoki samples were positive for *L. monocytogenes* in 2018 (Chen et al. 2018). The results of this research are generally consistent with compliance data generated through FDA import sampling.

*L. monocytogenes* may be present in the soil (Linke et al. 2014) and is common in natural and urban environments (Orsi and Wiedmann 2016). Contamination and proliferation of the pathogen are possible during cultivation, but also during harvesting and packing of enoki mushrooms (Gherman and Moran 2024). In fresh mushrooms, both *L. monocytogenes* and *Salmonella enterica* can survive and proliferate at 5°C, 10°C, and 25°C storage, as storage temperature does not inhibit growth (Fay, Salazar, George, et al. 2023). In mushrooms that are heat‐treated to inactivate pathogens, both pathogens exhibited a tailing effect and were not fully eliminated from enoki mushrooms when treated with 70°C, 80°C, or 90°C dry heat (Salazar et al. 2023). In dried mushrooms, a similar tailing effect has been observed during a 6‐month storage period, where the pathogens were not fully eliminated from enoki mushrooms (Fay, Salazar, Chavda, et al. 2023). *L. monocytogenes* and *S. enterica* are not fully eliminated by low moisture, dry heat, or refrigerated storage temperatures in fresh enoki, freshly dried enoki, and dried enoki stored long‐term. These characteristics make the control of *L. monocytogenes* in enoki mushrooms a challenge, as they allow for the growth and proliferation of pathogens, even though drying and reduced temperatures are typically used as controls for bacterial pathogens.

The potential for *L. monocytogenes* to contaminate enoki cultures during production has also been evaluated, and the research showed that *L. monocytogenes* could readily contaminate these cultures at any point during enoki cultivation (Grocholl et al. 2024). Enumeration data suggested that *L. monocytogenes* rapidly reached the carrying capacity of the substrate media and sustained that level, as similar levels of *L. monocytogenes* were observed regardless of how early or late in the culture process *L. monocytogenes* was added. In addition, data showed *L. monocytogenes* could persist in the substrate in the absence of enoki but be undetected until after enoki were added to the substrate, suggesting that testing of the substrate in the absence of enoki may result in false negative results.

### 2.10 Consumer Food Handling Practices

Little data has been collected during outbreak investigations in the United States to understand ill people's consumption and handling practices of enoki mushrooms. This is due to challenges with follow‐up and recall of what ingredients could be in dishes using enokis (Pereira et al. 2023; Kirchner et al. 2025). Ill people in listeriosis outbreaks linked to enoki mushrooms have reported consuming enoki mushrooms in hotpot and spring rolls; however, it is unknown how the spring roll containing enoki mushrooms was prepared and consumed. Enumeration results suggest that enoki mushrooms can be highly contaminated, making elimination a challenge and could have contributed to cross‐contamination in the home or in restaurants (Pereira et al. 2023; Kirchner et al. 2025). Research to determine the necessary time and temperature parameters for cooking enoki mushrooms to reduce the risk of *L. monocytogenes* in general and in various soup broths could aid the direction of safe handling, prevention, and educational efforts (Salazar et al. 2024).

Since adequate cooking kills *L. monocytogenes*, it is important to understand the method of preparation and cooking of enoki mushrooms, which would vary by dish. A common dish that contains enoki mushrooms is hotpot, an East Asian approach to cooking food in a communal simmering pot of flavored soup stock at the table, either at home or in restaurants. The heat source for the hotpot is placed directly on the dining table, and a variety of ingredients are served beside the pot for the diners to place into the hot soup themselves. Raw ingredients, such as thinly sliced meat, seafood, vegetables, mushrooms (including enoki), tofu, and dumplings, are placed into the soup, scooped out, and then more ingredients are added to the hotpot to replace them. Because diners are cooking the raw food themselves, consumers (as well as retail food establishments) would benefit from guidance regarding the broth temperatures and contact time necessary to be considered a kill step for each type of raw ingredient, such as enoki mushrooms. Until such conditions can be determined, there is the possibility of undercooking, since many times the source of heat may not be consistent, or target temperatures and/or necessary contact time may not be reached during preparation. In addition, there is a possibility of cross‐contamination since all raw foods are placed on the table.

Anecdotally, investigators in the United States found enokis are sometimes served raw, such as a garnish or lightly cooked at the end of preparation in a hotpot or other similar dishes. However, the FDA Food Code does not establish a time/temperature combination for cooking enoki mushrooms, and there are no specifications or standards on the apparatus used for the heating or cooking element. In other countries, however, enokis are customarily served fully cooked, and some packaging suggests enokis be cooked prior to consumption (Government of Canada 2023a, 2023b, 2023f). This information provides insight into the potential differences associated with the consumption of raw specialty mushrooms across varying geographical regions. As stated in the FDA's Strategy to Help Prevent Listeriosis and Salmonellosis Outbreaks Associated with Imported Enoki and Imported Wood Ear Mushrooms (U.S. Food and Drug Administration 2022), the FDA hopes to better understand routes of contamination and promote awareness of best practices to minimize contamination.

In a 2024 Porter Novelli study using an online survey of 1011 US adults that used quota sampling to obtain a nationally representative sample, 19% of respondents reported eating enoki mushrooms in the past 12 months. Among people who had eaten enoki mushrooms, 38% reported eating them at home, and 64% reported eating them away from home in the past month. About 24% of people who reported eating enoki mushrooms at home and 39% of people who reported eating enoki mushrooms away from home reported eating them raw or after a cooking method that would not completely inactivate *L. monocytogenes* (e.g., adding mushrooms to soup once the heat was turned off) (unpublished data).

There does not appear to be any scientifically rigorous assessment of how domestic retail establishments and consumers use enoki mushrooms; however, recipe sites and cooking blogs offer insight into how consumers consume or handle them. In a review of websites suggesting the use or consumption of enoki mushrooms, different recipes called for both raw and cooked enoki mushrooms. One site explicitly warned consumers to cook enoki mushrooms (“Tip: Enoki mushrooms are **not supposed to be eaten raw** [emphasis in original].”) (Red House Spice 2021). Several other recipes on well‐known sites, however, encourage consumption of raw enoki mushrooms (Food.com 2023a, 2023b, 2023c; Tsai 2023). One mushroom trade association publication also suggested trying raw enokis in a salad (Mushroom Council 2021).

International prevention advice has shifted since February 2023, when the Singapore Food Agency published recommended food safety practices for handling and consuming enoki mushrooms, which stated: “DO NOT eat raw enoki mushrooms”. This, along with correct storage and handling procedures to avoid cross‐contamination, was the first message for proper food safety practices (Quan 2023). The Food Standards of Australia and New Zealand and the United Kingdom Food Standards Authority posted similar advice in June and October 2023, respectively, after recalls of imported enoki mushrooms (Food Standards Australia and New Zealand 2023; United Kingdom Food Standards Agency 2023). Countries including the United Kingdom, Singapore, Korea, Canada, New Zealand, and Australia have issued prevention guidance regarding enoki mushrooms and *Listeria* spp. due to the increase in enoki mushroom‐related cases of listeriosis (Food Standards Australia and New Zealand 2023; Quan 2023; United Kingdom Food Standards Agency 2023; Government of Canada 2024). Ongoing work is being done in countries that are large producers of enoki mushrooms to better understand how to decrease *Listeria* spp. prevalence (Yoon et al. 2020; Kim et al. 2020).

During outbreak investigations, enoki mushrooms received by the United States from China and Korea were confirmed as the source of illnesses. Given the broad distribution of enoki mushrooms, sporadic illnesses linked to enoki mushrooms are likely not captured. CDC utilizes data platforms, such as PulseNet International and the European Centre for Disease Prevention and Control (ECDC) EpiPulse, to inquire if countries have isolates matching outbreak strains in the United States. Sharing WGS data through a public database, such as NCBI or PulseNet International, allows for international collaboration on cluster detection, development of hypotheses, and enhanced outbreak responses. This collaboration and data sharing have led to solving outbreaks linked to enoki mushrooms (Pettengill et al. 2020).

### 2.11 FDA Regulatory Activities: Outbreak and Non‐Outbreak Associated Recalls

Between March 2020 and October 2023, 27 recalls of enoki mushrooms were initiated in the United States, largely in response to state sampling efforts at retail of imported enoki mushrooms. FDA determined by entry declaration, labeling, traceback, and/or WGS that ten recalls were attributed to enoki mushrooms imported from facilities located in Korea, 12 recalls from facilities located in China, and four recalls from facilities located in Taiwan. Similarly, recurring recalls attributed to *L. monocytogenes* contamination of enoki mushrooms in Canada have been documented as recently as 2023 (Government of Canada 2019, 2020, 2023a, 2023b, 2023c, 2023d, 2023e, 2023f).

The FDA created the Import Alert system to provide uniform coverage and prevention of potentially violative products from being distributed in the United States. There are different types of import alerts (U.S. Food and Drug Administration 2019b), including country/world or area‐wide and firm/product‐specific. At present, there are three Import Alerts available for subjecting adulterated enoki mushrooms to detention without physical examination: Import Alert #99‐23, “Detention Without Physical Examination of Produce Due to Contamination with Human Pathogens,” Import Alert #99‐35, “Detention Without Physical Examination of Fresh Produce that Appears to Have Been Prepared, Packed or Held Under Insanitary Conditions,” and Import Alert #25‐21, “Detention Without Physical Examination of Enoki Mushrooms from Korea and China Due to *Listeria Monocytogenes*.”

Firms and their enoki mushrooms may be placed on one or both Import Alert #99‐23 and Import Alert #99‐35. If an FDA or state sample is found to be contaminated with a human pathogen, the firm and product would be subject to placement on the Red List of Import Alert #99‐23. If the FDA has gathered analytical evidence of a resident pathogen identified by sample collection, inspectional evidence, and/or epidemiologic and traceback evidence, then the firm and its enoki mushrooms would be subjected to placement on the Red List of Import Alert #99‐35. Firms subject to detention without physical examination per Import Alert #99‐23 and/or #25‐21 may submit a private laboratory analytical package for an entry of enoki mushrooms offered for import into the United States. To overcome the appearance of adulteration and obtain release, the product must be found to be non‐violative. In contrast, once a firm and its enoki mushrooms are placed on Import Alert #99‐35, a firm may not import into the United States without first providing evidence that the firm has resolved the conditions that gave rise to the appearance of the violation. The purpose of providing evidence is to ensure that the FDA will have confidence that future entries will follow the U.S. Federal Food, Drug, and Cosmetic Act. Enoki mushrooms imported from firms located in Korea and China may be detained without physical examination when offered for entry into the United States per Import Alert #25‐21, except for those that have successfully petitioned for removal from being subjected to Detention Without Physical Examination and been placed on the Green List of the import alert.

The FDA began increased surveillance sampling in early 2020 in response to the first outbreak of *L. monocytogenes* linked to enoki mushrooms. These sampling efforts led to the placement of 40 firms located in China, Japan, Korea, and Taiwan on the Red List of Import Alert #99‐23 and 20 firms located in China, Korea, and Taiwan on the Red List of Import Alert #99‐35. Table 3 illustrates the addition of the enoki mushrooms from firms located in China, Korea, and Taiwan to the Red List of the identified Import Alerts, over this timeframe.

**TABLE 3:** The addition of enoki mushrooms from firms located in the People's Republic of China, the Republic of Korea, and Taiwan to the Red List of the identified Import Alerts by Fiscal Year (FY).

	FY2020	FY2021	FY2022	FY2023	Cumulative FY20‐23
People's Republic of China	0	1	11	11	23
Republic of Korea	5	10	6	0	21
Taiwan	0	0	5	10	15

Based on FDA and state sampling, WGS, traceback and epidemiological evidence associated with both confirmed outbreaks of *L. monocytogenes* linked to enoki mushrooms, and engagement with the competent authorities in Korea and China, FDA established Import Alert #25‐21 with the addition of enoki mushrooms from Korea on July 1, 2022, and enoki mushrooms from China on March 1, 2023.

From October 2020 to July 2023, the FDA collected approximately 316 samples out of 9907 entry lines of imported enoki mushrooms from facilities located in Canada, China, Japan, Korea, and Taiwan. *Listeria* spp. were detected in 184 of the 316 (58%) samples, with *L. monocytogenes* found in 58 (18%) samples. *L. monocytogenes* was isolated in 24 of 95 (25%) samples of enoki mushrooms imported from facilities located in Korea, 20 out of 149 (13%) samples imported from China, 12 of 41 (29%) samples imported from Taiwan, and 2 of 24 (8%) samples imported from Japan. *L. monocytogenes* has not been isolated in the seven samples collected from firms located in Canada, which is consistent with reports from CFIA that routine sampling and testing of their domestic Canadian production of enoki have consistently tested negative for *L. monocytogenes*. Other *Listeria* spp. detected in FDA samples include *L. innocua*, *L. grayi, L. welshimeri, L. seeligeri*, *and L. ivanovii*. Due to selective growth advantages and inhibitory interactions, *L. innocua* is known to outcompete *L. monocytogenes* in various types of enrichment media, including the Buffered Listeria Enrichment Broth (BLEB) used in the FDA enrichment protocol (Hitchins et al. 2022). Thus, the dual presence of *L. innocua* and *L. monocytogenes* in a sample significantly reduces the ability to detect the presence of *L. monocytogenes* even with the use of chromogenic agar media (Keys et al. 2013; Cornu et al. 2002).

### 2.12 Laboratory Flexible Funding Model Enoki Listeria Surveillance

From July 2021 to December 2024, state partners collected and analyzed 585 samples of enoki mushrooms as part of the Laboratory Flexible Funding Model (LFFM) Cooperative Agreement (U.S. Food and Drug Administration 2023c). Ten (10) participating state agencies collected and analyzed enoki mushroom samples, including California Department of Public Health, Hawaii Department of Health, Maryland Department of Health, Michigan Department of Agriculture and Rural Development, Missouri Department of Health and Senior Services, New Hampshire Department of Health and Human Services, South Carolina Department of Health and Environmental Control, Tennessee Department of Agriculture, University of Iowa State Hygienic Laboratory, and West Virginia Department of Agriculture. Per review of the label, sampled enoki mushrooms originated from Canada (127 of 585, 22%), China (155 of 585, 26%), Japan (12 of 585, 2%), Korea (179 of 585, 31%), Taiwan (27 of 585, 5%), United States (41 of 585, 7%), and unknown or no country indicated on the label (44 of 585, 8%). All samples were collected at retail grocery stores. The first enoki samples labelled as “USA grown” were collected in 2024.

*Listeria* spp. were identified in 99 of 585 (17%) of samples, and *L. monocytogenes* was isolated in 45 of 585 (8%) of samples. Enumeration was performed on 30 of the 45 *L. monocytogenes* positive samples using MPN and direct plating enumeration methods. Results ranged from <0.03 to 430,000 MPN/g and 50 to 200,000 CFU/g. *L. grayi* was detected in 31 of 585 (5%) of samples, *L. innocua* was detected in 73 of 585 (12%) of samples, and *L. seeligeri* was detected in 1 of 585 (<1%) of samples; 15 of the 99 (15%) samples positive for one or more *Listeria* spp. were also positive for *L. monocytogenes*. All confirmed *L. monocytogenes* samples had at least one *L. monocytogenes* isolate sequenced. A total of 168 *L. monocytogenes* and *Listeria* spp. isolates were sequenced and uploaded to NCBI.

### 2.13 Actions Resulting From State LFFM Enoki Samples

As of the end of calendar year 2024, a total of 15 recalls of enoki mushrooms have occurred in the United States because of LFFM sampling (Table 1). Seven (7) consumer advisories have been issued by the FDA and five state agencies (Table 1). Notably, one recall of enoki mushrooms for contamination with *L. monocytogenes* was linked to a domestic grower in October 2024 (U.S. Food and Drug Administration 2024a).

Two foreign firms (located in China) on Import Alert 99‐23 for other products had enoki mushrooms added based on LFFM sampling (U.S. Food and Drug Administration 2021). Four firms (located in China [2] and Taiwan [2]) were added to Import Alert 99‐23 based on FDA import positive samples collected as a follow‐up to the LFFM sampling or LFFM samples (U.S. Food and Drug Administration 2021). Three firms (located in China) were added to Import Alert 99‐35 based on FDA import and LFFM samples (U.S. Food and Drug Administration 2021). LFFM samples were utilized (along with FDA samples) to support the country‐wide import alert for enoki mushrooms from Korea, Import Alert 25‐21, and later expanded to enoki mushrooms from China (U.S. Food and Drug Administration 2023b). One firm (located in Korea) was removed from the green list of Import Alert 25‐21 based on LFFM samples (U.S. Food and Drug Administration 2023b).

### 2.14 LFFM Enoki Samples Related to Outbreak Investigations

A routine LFFM sample of enoki mushrooms collected by the Missouri Department of Health and Senior Services in late November 2022 was positive for *L. monocytogenes*. The sample resulted in an FDA‐issued safety alert on December 17, 2022 (U.S. Food and Drug Administration 2023a). WGS later revealed that the strain of *Listeria* found in the sample matched one of two outbreak strains in the 2022 multi‐state listeriosis outbreak linked to enoki mushrooms (U.S. Food and Drug Administration 2023d). In early January 2023, the Maryland Department of Health identified *L. monocytogenes* in two routine LFFM samples of enoki mushrooms with the same labeling as the Missouri sample. WGS revealed that both outbreak strains were present in the Maryland samples, and it became clear that the enoki mushrooms sampled by Maryland and Missouri were part of a distribution chain related to the 2022 outbreak, but not covered by the December 13, 2022, or January 13, 2023, firm recalls (U.S. Centers for Disease Control and Prevention 2023b; U.S. Food and Drug Administration 2023d). While neither Maryland nor Missouri had cases in the outbreak, their positive product samples helped confirm the traceback investigation and source of the outbreak. These samples serve as a good example of how surveillance sampling can support outbreak investigations.

### 2.15 Challenges in Enoki Outbreak Investigations: Epidemiology

Genomic data from sampling activities spurred further investigation into enoki mushrooms as a leading hypothesis linked to the illnesses in both US outbreaks linked to enoki mushrooms (Pereira et al. 2023; Kirchner et al. 2025). The associated samples provided valuable information to help guide the epidemiologic investigation into an unusual food item that was not initially reported by ill people or their surrogates. Surrogate interviews are conducted when the patient is too ill or has passed away. These interviews may be challenging because surrogates are often family members of the patient who are caring for or grieving their loved one. These interviews can also be less detailed if the surrogate did not live with or provide food for the patient. Without product sampling and WGS data from isolates, identifying enoki mushrooms as the source of illness may not have occurred for any outbreak investigation for several reasons. First, general fresh mushrooms are a food listed on the standard LI case report form (U.S. Centers for Disease Control and Prevention 2023a), which requires a patient or surrogate to provide details about the types of fresh mushrooms consumed in the 28 days prior to their illness onset. The LI form does not explicitly include a question about enoki mushrooms for investigators to ask, so often they must conduct a second interview using a supplemental questionnaire to directly ask an ill person or their surrogate additional details about enoki mushroom exposures. At times, ill people or their surrogates are not willing to complete additional interviews, and mushroom details are unavailable.

Food recalls can be further hindered due to the timeframe between potential exposure dates and when an interview is conducted, which can range from weeks to months. In outbreaks thus far, only three ill people reported enoki mushrooms when they were specifically asked, and one person specified enoki mushrooms on their initial interview. Next, enoki mushrooms can be served as a garnish or act as a stealth ingredient in dishes, meaning they may not be identified by ill people even when asked directly because they may not recognize or remember that one ingredient in a complex multi‐ingredient dish. Finally, enoki mushrooms may be labeled under other names, further complicating their identification by ill people. Since the first outbreak, investigators developed specific interview questions targeting enoki mushroom consumption and employed tactics such as reviewing menus and photos at restaurants to gather insights as to whether a patient could have consumed enoki mushrooms prior to their illness. Photos of enoki mushrooms are also sometimes provided to ill people during interviews to aid in the identification of the mushrooms.

### 2.16 Traceback

There were multiple challenges for traceback during the previously discussed enoki outbreak investigations, ranging from issues specific to these two outbreaks to more basic traceback challenges that have been previously described in the literature (Pereira et al. 2023; Kirchner et al. 2025). Lack of specific exposure data limited traceback capabilities, as there was not enough exposure information for enough people. The 2020 outbreak investigation included illnesses from 2016, so contacting people ill in previous years was not possible, and therefore, investigators had to traceback enoki mushroom samples matching the outbreak strain (Pereira et al. 2023). Similarly, in the 2022 outbreak, the majority of the traceback was conducted on samples that matched the outbreak strains or with very basic exposure information for three people. The lack of record keeping and interoperability along the supply chain was another hindrance because a US importer was unable to decipher the traceability system used by the foreign firm to determine which Korean manufacturer supplied the contaminated enoki mushrooms. All enoki mushrooms linked to the cases in the United States and Canada were imported; however, the FDA cannot inspect foreign companies as readily as domestic companies, and their supply chains can be quite complex, which can introduce complications when a robust traceability system is not implemented (Pereira et al. 2023). Language inconsistencies, a lack of confidentiality commitment agreements, and diplomatic considerations prevented the FDA from reaching out directly to foreign manufacturers to resolve traceback issues in a timely fashion during the 2020 outbreak investigation. During the 2022 outbreak investigation, the US importers and distributors utilized a box‐in‐box‐out method for stock rotation and rarely opened the boxes to inspect the product to ensure it matched the expected product description. This resulted in confusion along the supply chain regarding exactly what product was received and the supplier from whence it came. This issue was underscored when a US importer/distributor claimed that a product positive for the outbreak strains of *L. monocytogenes* was incorrectly labeled as being from their company and was instead from another foreign supplier. Therefore, with a lack of insight into the supply chain of the product, a definitive conclusion could not be reached.

### 2.17 Prevention Strategy

The FDA launched a series of prevention strategies to reduce the public health impact of foodborne illness associated with the consumption of certain FDA‐regulated foods (U.S. Food and Drug Administration 2022). Development of these prevention strategies includes an examination of potential sources and routes of contamination for commodity–pathogen pairings and design initiatives involving disciplines such as investigative and compliance action, research, education, and outreach activities for the enoki mushroom growing industry.

In May 2021, the FDA implemented a strategy to help prevent future outbreaks linked to imported enoki mushrooms (U.S. Food and Drug Administration 2022). The strategy includes four key measures. First, ensuring exporting firms and competent authorities are aware of the applicable PSR requirements for specialty mushrooms and the potential differences associated with the consumption of raw specialty mushrooms. This is intended to help international growers understand that their product may be consumed without a kill step by US consumers, as opposed to the preparation practices used in their countries. Second, engaging food safety authorities in Korea, China, Canada, Taiwan, and Japan to better understand industry production practices or other contributing factors for potential sources of contamination in enoki mushrooms, and what producers are doing to prevent contamination of these mushrooms. Third, conducting research to better understand *L. monocytogenes* contamination mechanisms and survival on enoki mushrooms. Finally, increasing strategic and targeted FDA sampling of imported specialty mushrooms at US ports of entry and domestically at US retail operations.

FDA is continually optimizing its border surveillance program by targeting the highest‐risk products for sampling at a port of entry, which is designed to prevent contaminated products from entering domestic commerce. For example, when specific producers are linked to a contaminated shipment, the FDA may also detain future shipments until the producer can assure the FDA that the conditions that gave rise to the violation have been resolved. Along with targeted FDA sampling, increasing state sampling of specialty mushrooms at retail operations in the United States can be used to identify mushrooms that may be contaminated and already available to consumers. These activities could result in potentially contaminated products being recalled and removed from the market. The FDA will work with industry to ensure international specialty mushroom producers have access to training intended to help them meet requirements for ensuring the safe production of mushrooms for import into the U.S.

In addition to interactions with food safety competent authorities in listeriosis outbreak investigations in 2020–2022 with Korea and in 2023 with China, FDA initiated a series of government‐to‐government engagements in 2021 with counterparts in Korea, China, Japan, Taiwan, and Canada, as part of FDA's program for Prevention Strategies to Enhance Food Safety (U.S. Food and Drug Administration 2024b). As mentioned on the program's website, “*A prevention strategy is an affirmative, deliberate approach undertaken by the FDA and interest holders to address the root cause or likely causal factors of outbreaks linked to certain FDA‐regulated foods and help limit or prevent future outbreaks. These strategies are not developed for every outbreak. They generally target scenarios where the likely root cause or contributing factors are more clearly identified and similar operations or historical outbreak patterns suggest a repeated risk* (U.S. Food and Drug Administration 2025).” As part of this program, on September 6, 2022, the FDA published a “Strategy to Help Prevent Listeriosis and Salmonellosis Outbreaks Associated with Imported Enoki and Imported Wood Ear Mushrooms” (U.S. Food and Drug Administration 2022). The activities in this strategy helped provide focus on the type of engagement and collaboration pursued with FDA counterparts. These activities include technical meetings to share information on the food safety event at hand, learning from government, and, in some cases, directly from industry as well, on industry production practices or other factors that may have contributed to the contamination, and activities being considered or undertaken by industry or government to help prevent contamination in the future. As part of identifying potential preventive measures, the FDA and other partners have been conducting research on the presence, growth, and survival of *L. monocytogenes* on enoki mushrooms, and cleaning, sanitation, and sterilization during production and holding operations. In addition, the collaboration has helped identify industry educational needs, including required food safety training as required for compliance with the FDA's Produce Safety Rule (PSR) (See 21 CFR Part 112: Standards for the Growing, Harvesting, Packing, and Holding of Produce for Human Consumption) (U.S. Food and Drug Administration 2020). As of the publication of this article, engagement with counterparts in China and Korea continues as a part of this prevention strategy initiative.

Through the successful completion of these activities, it is the FDA's intention to better understand the routes of contamination and promote awareness of best practices to minimize contamination, ensure broad industry awareness of applicable food safety regulations, continue to encourage and support research efforts, and prevent future illness associated with the consumption of contaminated enoki mushrooms.

### 2.18 Labeling

As discussed above, mushrooms are generally a covered commodity under the PSR 21 CFR Part 112: Standards for the Growing, Harvesting, Packing, and Holding of Produce for Human Consumption. All activities associated with the cultivation of enoki mushrooms in covered operations are required to comply with applicable requirements in the PSR. The PSR's standards are designed to reduce the potential for microbial contamination that may be introduced to any commodity through growing, harvesting, packing, and holding activities. Neither the PSR nor food labeling regulations in 21 CFR Part 101 require mushroom producers to include information on safe handling and preparation of mushrooms. However, the FDA does not object to the voluntary addition of label information regarding safe handling instructions, provided it is truthful and not misleading.

## 3 Conclusions

Outbreaks linked to imported enoki mushrooms contaminated with *L. monocytogenes* provided further evidence to the emerging and persisting issue of foodborne illnesses linked to this commodity–pathogen pair. In addition to epidemiologic and traceback evidence, genomic data from historical human isolates and isolates recovered from enoki mushroom samples, along with international data sharing, assisted investigators in identifying the source of these outbreaks. These investigations and sampling findings resulted in numerous regulatory actions to protect public health. These actions include product recalls, import actions, and increased surveillance sampling for enoki mushrooms. Evaluation of the information gathered during these investigations revealed the need to gain a better understanding of how *L. monocytogenes* contaminates enoki mushrooms and persists in the product. Current research suggests that a significant portion of US consumers who consume enoki mushrooms are likely to eat them either raw or inadequately cooked. Opportunities to partner with industry and international food safety authorities are important to understand current manufacturing practices and to ensure mushroom producers are aware of their responsibility to produce safe food, as the FDA will refuse products where pathogenic contamination is indicated. Through the ongoing activities of the FDA's prevention strategy, enhanced surveillance processes, and international and industry engagement, public health and regulatory agencies are taking actions to reduce the burden of foodborne disease associated with the consumption of enoki mushrooms.

## Author Contributions

**Stelios Viazis**: conceptualization, writing – original draft, writing – review and editing, project administration, supervision. **Amanda Conrad**: conceptualization, writing – original draft, writing – review and editing, investigation. **Alexandra Palacios**: writing – original draft, writing – review and editing, investigation. **Alexandra L. Busbee**: writing – original draft, writing – review and editing, investigation. **Margaret Kirchner**: writing – original draft, writing – review and editing, investigation. **Natalie Cataldo**: writing – original draft, writing – review and editing, investigation. **Allison Wellman**: writing – original draft, writing – review and editing, investigation. **Brett Weed**: writing – original draft, writing – review and editing. **Christina K. Carstens**: writing – original draft, writing – review and editing. **Victoria Wagoner**: writing – review and editing. **Laurel S. Burall**: writing – review and editing, writing – original draft. **Joelle K. Salazar**: writing – review and editing, writing – original draft. **Megan L. Fay**: writing – original draft, writing – review and editing. **Kenneth Nieves**: writing – review and editing. **Zachary Miller**: writing – review and editing. **David T. Ingram**: writing – review and editing. **Stephen Hughes**: writing – review and editing, writing – original draft. **Joseph Baugher**: writing – review and editing. **Robert Hatch**: writing – review and editing. **Scott Izyk**: writing – review and editing. **Grace Pederson**: writing – review and editing. **Allison Scott**: writing – review and editing. **Matthew C. Kristof**: writing – review and editing. **Laurie Williams**: writing – review and editing. **Lauren Yeung**: writing – review and editing, writing – original draft, data curation. **Cary Chen Parker**: project administration, writing – review and editing, supervision. **Michael Bazaco**: writing – original draft, writing – review and editing, conceptualization.

## Disclosure

The findings and conclusions of this report are those of the authors and do not necessarily represent the official position of the Centers for Disease Control and Prevention (CDC) or the Food and Drug Administration (FDA).

## Conflicts of Interest

The authors declare no conflicts of interest.

## Note

1 Taiwan is listed as “Other Asia, not elsewhere specified” in official World Bank trade reports.

## References

[crf370292-bib-0001] Chen, M. , J. Cheng , Q. Wu , et al. 2018. “Prevalence, Potential Virulence, and Genetic Diversity of *Listeria monocytogenes* Isolates from Edible Mushrooms in Chinese Markets.” Frontiers in Microbiology 9: 1711. 10.3389/fmicb.2018.01711. 30100901 PMC6072871

[crf370292-bib-0002] Cornu, M. , M. Kalmokoff , and J. P. Flandrois . 2002. “Modelling the Competitive Growth of *Listeria monocytogenes* and *Listeria Innocua* in Enrichment Broths.” International Journal of Food Microbiology 73, no. 2–3: 261–274. 10.1016/s0168-1605(01)00658-4. 11934034

[crf370292-bib-0003] Crosby, W. 2019. Speciality Mushroom Cultivation in the Northeast United States. Willie Crosby & Fungi Ally LLC. https://smallfarms.cornell.edu/wp‐content/uploads/2020/04/Specialty_Mushrooms_BookMC2.pdf.

[crf370292-bib-0004] Davis, S. , J. B. Pettengill , Y. Luo , et al. 2015. “CFSAN SNP Pipeline: An Automated Method for Constructing SNP Matrices from Next‐Generation Sequence Data.” PeerJ Computer Science 1: e20. 10.7717/peerj-cs.20.

[crf370292-bib-0005] Dong, Z. , Y. Xiao , and H. Wu . 2021. “Selenium Accumulation, Speciation, and Its Effect on Nutritive Value of *Flammulina velutipes* (Golden Needle Mushroom).” Food Chemistry 350: 128667. 33288349 10.1016/j.foodchem.2020.128667

[crf370292-bib-0006] Fay, M. L. , J. K. Salazar , N. J. Chavda , G. R. Patil , and D. T. Ingram . 2023. “Survival Kinetics of *Listeria monocytogenes* and *Salmonella enterica* on Dehydrated Enoki and Wood Ear Mushrooms During Long‐Term Storage.” Food Microbiology 114: 104304. 37290867 10.1016/j.fm.2023.104304

[crf370292-bib-0007] Fay, M. L. , J. K. Salazar , J. George , et al. 2023. “Modeling the Fate of *Listeria monocytogenes* and *Salmonella enterica* on Fresh Whole and Chopped Wood Ear and Enoki Mushrooms.” Journal of Food Protection 86, no. 5: 100075. 36989858 10.1016/j.jfp.2023.100075

[crf370292-bib-0008] Food Standards Australia and New Zealand . 2023. “ *Listeria monocytogenes* and Imported Fresh Enoki Mushrooms.” Published November 7. https://www.foodstandards.gov.au/consumer/generalissues/Pages/Listeria‐Monocytogenes‐linked‐to‐fresh‐enoki‐mushrooms‐imported‐from‐South‐Korea.aspx.

[crf370292-bib-0009] Food.com . 2023a. “Citrus Salad With Shiitake and Enoki Mushrooms.” Accessed December 1. https://www.food.com/recipe/citrus‐salad‐with‐shiitake‐and‐enoki‐mushrooms‐405245.

[crf370292-bib-0010] Food.com . 2023b. “Cucumber and Enoki Salad.” Accessed December 1. https://www.food.com/recipe/cucumber‐enoki‐salad‐16643.

[crf370292-bib-0011] Food.com . 2023c. “Marinated Enoki Salad.” Accessed December 1. https://www.food.com/recipe/marinated‐enoki‐salad‐345084.

[crf370292-bib-0012] Fotopoulou, E. T. , C. Jenkins , A. Painset , and C. Amar . 2024. “ *Listeria monocytogenes*: The Silent Assassin.” Journal of Medical Microbiology 73, no. 3: 001800. 38506266 10.1099/jmm.0.001800 PMC10963902

[crf370292-bib-0013] Ge, Z.‐W. , X.‐B. Liu , K. Zhao , and Z.‐L. Yang . 2015. “Species Diversity of *Flammulina* in China: New Varieties and a New Record.” Mycosystema 34, no. 4: 589–603.

[crf370292-bib-0014] Gherman, I. , and K. Moran . 2024. “Rapid Risk Assessment of the Detection of *Listeria monocytogenes* in Enoki Mushrooms.” FSA Research and Evidence. Published September 10. 10.46756/001c.122719.

[crf370292-bib-0016] Government of Canada . 2019. “Notification—Jongilpoom Brand Enoki Mushrooms Recalled Due to *Listeria monocytogenes* .” Accessed March 5. https://www.inspection.gc.ca/food‐recall‐warnings‐and‐allergy‐alerts/2019‐07‐05‐r13072/eng/1563312477630/1563312479417.

[crf370292-bib-0017] Government of Canada . 2020. “Food Recall Warning—Golden Mushroom Brand Enoki Mushroom Recalled Due to *Listeria monocytogenes* .” Accessed January 6. https://inspection.canada.ca/food‐recall‐warnings‐and‐allergy‐alerts/2020‐03‐24/eng/1585081415872/1585081416327.

[crf370292-bib-0018] Government of Canada . 2023a. “Golden Mushroom Brand Enoki Mushroom Recalled due to *Listeria monocytogenes* .” Updated September 16. https://recalls‐rappels.canada.ca/en/alert‐recall/golden‐mushroom‐brand‐enoki‐mushroom‐recalled‐due‐listeria‐monocytogenes‐2?utm_source=gc‐notify&utm_medium=email&utm_content=en&utm_campaign=hc‐sc‐rsa‐22‐23.

[crf370292-bib-0019] Government of Canada . 2023b. “Good Brand Enoki Mushroom Recalled Due to *Listeria monocytogenes* .” Updated December 6. https://recalls‐rappels.canada.ca/en/alert‐recall/good‐brand‐enoki‐mushroom‐recalled‐due‐listeria‐monocytogenes?utm_source=gc‐notify&utm_medium=email&utm_content=en&utm_campaign=hc‐sc‐rsa‐22‐23.

[crf370292-bib-0020] Government of Canada . 2023c. “Lian Teng Brand "Champignon Énoki" (Enoki Mushrooms) Recalled Due to *Listeria monocytogenes* .” Updated October 25. https://recalls‐rappels.canada.ca/en/alert‐recall/lian‐teng‐brand‐champignon‐enoki‐enoki‐mushrooms‐recalled‐due‐listeria‐monocytogenes?utm_source=gc‐notify&utm_medium=email&utm_content=en&utm_campaign=hc‐sc‐rsa‐22‐23.

[crf370292-bib-0021] Government of Canada . 2023d. “O'Ya Hoho Brand 100% Fresh Enoki Mushrooms Recalled Due to *Listeria monocytogenes* .” Updated November 21. https://recalls‐rappels.canada.ca/en/alert‐recall/o‐ya‐hoho‐brand‐100‐fresh‐enoki‐mushrooms‐recalled‐due‐listeria‐monocytogenes?utm_source=gc‐notify&utm_medium=email&utm_content=en&utm_campaign=hc‐sc‐rsa‐22‐23.

[crf370292-bib-0022] Government of Canada . 2023e. “SSS Brand Mushroom (Enoki) Recalled due to *Listeria monocytogenes* .” Updated July 28. https://recalls‐rappels.canada.ca/en/alert‐recall/sss‐brand‐mushroom‐enoki‐recalled‐due‐listeria‐monocytogenes?utm_source=gc‐notify&utm_medium=email&utm_content=en&utm_campaign=hc‐sc‐rsa‐22‐23.

[crf370292-bib-0023] Government of Canada . 2023f. “Super Brand Enoki Mushroom Recalled due to *Listeria monocytogenes* .” Updated September 19. https://recalls‐rappels.canada.ca/en/alert‐recall/super‐brand‐enoki‐mushroom‐recalled‐due‐listeria‐monocytogenes?utm_source=gc‐notify&utm_medium=email&utm_content=en&utm_campaign=hc‐sc‐rsa‐22‐23.

[crf370292-bib-0024] Government of Canada . 2024. “Imported Raw Enoki Mushrooms: What You Need to Know For Safe Food Practices.” Updated September 10. https://recalls‐rappels.canada.ca/en/alert‐recall/imported‐raw‐enoki‐mushrooms‐what‐you‐need‐know‐safe‐food‐practices.

[crf370292-bib-0025] Grocholl, J. , M. Ferguson , S. Hughes , S. Trujillo , and L. S. Burall . 2024. “ *Listeria monocytogenes* Contamination Leads to Survival and Growth During Enoki Mushroom Cultivation.” Journal of Food Protection 87, no. 6: 100290. 38701973 10.1016/j.jfp.2024.100290

[crf370292-bib-0026] Hitchins, A. D. , K. Jinneman , and Y. Chen . 2022. BAM Chapter 10: Detection of Listeria monocytogenes in Foods and Environmental Samples, and Enumeration of Listeria monocytogenes in Foods. U.S. Food & Drug Administration.

[crf370292-bib-0027] Interagency Food Safety Analytics Collaboration . 2024. “Foodborne Illness Source Attribution Estimates—United States, 2022.” Published December 13. https://www.cdc.gov/ifsac/php/data‐research/annual‐report‐2022.html#cdc_report_pub_study_section_2‐introduction.

[crf370292-bib-0028] Jackson, B. R. , C. Tarr , E. Strain , et al. 2016. “Implementation of Nationwide Real‐Time Whole‐Genome Sequencing to Enhance Listeriosis Outbreak Detection and Investigation.” Clinical Infectious Diseases 63, no. 3: 380–386. 10.1093/cid/ciw242. 27090985 PMC4946012

[crf370292-bib-0029] Keys, A. L. , R. C. Dailey , A. D. Hitchins , and R. D. Smiley . 2013. “Postenrichment Population Differentials Using Buffered *Listeria* Enrichment Broth: Implications of the Presence of *Listeria innocua* on *Listeria monocytogenes* in Food Test Samples.” Journal of Food Protection 76, no. 11: 1854–1862. 10.4315/0362-028x.Jfp-13-089. 24215687 PMC5114845

[crf370292-bib-0030] Kim, S.‐R. , W.‐I. Kim , J.‐H. Yoon , et al. 2020. “Growth Survival of *Listeria monocytogenes* in Enoki Mushroom (*Flammulina velutipes*) at Different Temperatures and Antilisterial Effect of Organic Acids.” Journal of Food Hygiene and Safety 35, no. 6: 630–636.

[crf370292-bib-0031] Kirchner, M. , A. Palacios , N. Cataldo , et al. 2025. “A Binational Sample‐Initiated Retrospective Outbreak Investigation of *Listeria monocytogenes* Infections in the United States and Canada Linked to Enoki Mushrooms Imported From China 2022–2023.” Journal of Food Protection 88, no. 1: 100413. 10.1016/j.jfp.2024.100413. https://www.sciencedirect.com/science/article/pii/S0362028x24001972. 39571796

[crf370292-bib-0032] Lee, J. , K.‐W. Jeong , B. Jin , et al. 2013. “Structural and Dynamic Features of Cold‐Shock Proteins of *Listeria monocytogenes*, a Psychrophilic Bacterium.” Biochemistry 52, no. 14: 2492–2504. 10.1021/bi301641b. 23506337

[crf370292-bib-0033] Linke, K. , I. Rückerl , K. Brugger , et al. 2014. “Reservoirs of *Listeria* Species in Three Environmental Ecosystems.” Applied and Environmental Microbiology 80, no. 18: 5583–5592. 25002422 10.1128/AEM.01018-14 PMC4178586

[crf370292-bib-0034] Liu, D. , M. L. Lawrence , A. Jerald Ainsworth , and F. W. Austin . 2005. “Comparative Assessment of Acid, Alkali and Salt Tolerance in *Listeria monocytogenes* Virulent and Avirulent Strains.” FEMS Microbiology Letters 243, no. 2: 373–378. 15686837 10.1016/j.femsle.2004.12.025

[crf370292-bib-0035] Madad, A. , K. E. Marshall , T. Blessington , et al. 2023. “Investigation of a Multistate Outbreak of *Listeria monocytogenes* Infections Linked to Frozen Vegetables Produced at Individually Quick‐Frozen Vegetable Manufacturing Facilities.” Journal of Food Protection 86, no. 8: 100117. 10.1016/j.jfp.2023.100117. https://www.sciencedirect.com/science/article/pii/S0362028x23068011. 37327999 PMC10829048

[crf370292-bib-0036] Methven, A. , K. W. Hughes , and R. H. Petersen . 2000. “ *Flammulina* RFLP Patterns Identify Species and Show Biogeographical Patterns Within Species.” Mycologia 92, no. 6: 1064–1070.

[crf370292-bib-0037] Miller, A. J. , J. E. Call , and B. Shawn Eblen . 1997. “Growth, Injury, and Survival Potential of *Yersinia enterocolitica*. *Listeria monocytogenes*, and *Staphylococcus aureus* in Brine Chiller Conditions.” Journal of Food Protection 60, no. 11: 1334–1340. 31207768 10.4315/0362-028X-60.11.1334

[crf370292-bib-0038] Tsai, M. 2023. “Miso Broth with Tatsoi‐Enoki Salad.” Accessed December 1. https://www.foodnetwork.com/recipes/miso‐broth‐with‐tatsoi‐enoki‐salad‐recipe‐1918234.

[crf370292-bib-0039] Morgan, K. 2023. “Specialty Mushrooms are Booming – Including in Home Gardens.” Washington Post, April 13. https://www.washingtonpost.com/home/2023/04/13/edible‐mushrooms‐grow‐home/.

[crf370292-bib-0040] Mushroom Council . 2021. “Versatility in Varieties.” Accessed December 1. https://www.mushroomcouncil.org/wp‐content/uploads/2021/04/All‐About‐Mushrooms‐Kit.pdf.

[crf370292-bib-0041] Orsi, R. H. , and M. Wiedmann . 2016. “Characteristics and Distribution of *Listeria* spp., Including *Listeria* Species Newly Described Since 2009.” Applied Microbiology and Biotechnology 100: 5273–5287. 27129530 10.1007/s00253-016-7552-2 PMC4875933

[crf370292-bib-0042] Pereira, E. , A. Conrad , A. Tesfai , et al. 2023. “Multinational Outbreak of *Listeria monocytogenes* Infections Linked to Enoki Mushrooms Imported From the Republic of Korea 2016–2020.” Journal of Food Protection 86, no. 7: 100101. 10.1016/j.jfp.2023.100101. 37169291 PMC10947956

[crf370292-bib-0043] Pettengill, J. B. , A. Markell , A. Conrad , et al. 2020. “A Multinational Listeriosis Outbreak and the Importance of Sharing Genomic Data.” Lancet Microbe 1, no. 6: e233–e234. 10.1016/S2666-5247(20)30122-1. 35544215 PMC10966468

[crf370292-bib-0044] Pohl, A. M. , R. Pouillot , M. C. Bazaco , et al. 2019. “Differences Among Incidence Rates of Invasive Listeriosis in the U.S. FoodNet Population by Age, Sex, Race/Ethnicity, and Pregnancy Status, 2008–2016.” Foodborne Pathogens and Disease 16, no. 4: 290–297. 10.1089/fpd.2018.2548. 30735066 PMC6482898

[crf370292-bib-0045] Red House Spice . 2021. “Enoki Mushroom, How to Prepare and Cook.” Accessed December 1. https://redhousespice.com/steamed‐enoki‐mushroom/.

[crf370292-bib-0046] Salazar, J. K. , M. Fay , G. Fleischman , B. Khouja , D. S. Stewart , and D. T. Ingram . 2024. “Inactivation Kinetics of *Listeria monocytogenes* and *Salmonella enterica* on Specialty Mushroom Garnishes Based on Ramen Soup Broth Temperature.” Frontiers in Microbiology 15: 1485398. 39697650 10.3389/fmicb.2024.1485398 PMC11652501

[crf370292-bib-0047] Salazar, J. K. , M. L. Fay , B. A. Khouja , N. J. Chavda , G. R. Patil , and D. T. Ingram . 2023. “Effect of Dehydration on the Inactivation of *Listeria monocytogenes* and *Salmonella enterica* on Enoki and Wood Ear Mushrooms.” Frontiers in Microbiology 14: 1257053. 38029214 10.3389/fmicb.2023.1257053 PMC10644103

[crf370292-bib-0048] Scallan Walter, E. J. , Z. Cui , R. Tierney , et al. 2019. “Foodborne Illness Acquired in the United States–Major Pathogens.” Emerging Infectious Diseases 2025; 31, no. 4: 669–677. 10.3201/eid3104.240913. PMC11950263 40133035

[crf370292-bib-0049] Settanni, L. , and A. Corsetti . 2007. “The Use of Multiplex PCR to Detect and Differentiate Food‐ and Beverage‐Associated Microorganisms: A Review.” Journal of Microbiological Methods 69, no. 1: 1–22. 10.1016/j.mimet.2006.12.008. https://www.sciencedirect.com/science/article/pii/S0167701206003587. 17280731

[crf370292-bib-0050] Sharma, V. P. , A. Barh , R. K. Bairwa , S. K. Annepu , B. Kumari , and S. Kamal . 2021. “Enoki Mushroom (*Flammulina velutipes* (Curtis) Singer) Breeding.” In Advances in Plant Breeding Strategies: Vegetable Crops: Volume 10: Leaves, Flowerheads, Green Pods, Mushrooms and Truffles, edited by J. M. Al‐Khayri , S. M. Jain , and D. V. Johnson , 423–441. Springer.

[crf370292-bib-0051] Quan, T. Y. 2023. “Safe Practices when Handling and Consuming Enoki Mushrooms.” Singapore Food Agency. Published February 16. https://www.sfa.gov.sg/food‐information/risk‐at‐a‐glance/enoki‐mushrooms.

[crf370292-bib-0052] Tolar, B. , L. A. Joseph , M. N. Schroeder , et al. 2019. “An Overview of PulseNet USA Databases.” Foodborne Pathogens and Disease 16, no. 7: 457–462. 10.1089/fpd.2019.2637. 31066584 PMC6653802

[crf370292-bib-0053] Tonomura, H. 1978. “Flammulina velutipes.” In The Biology and Cultivation of Edible Mushrooms, edited by S. T. Chang and W. A. Hayes , 409–421. Academic Press.

[crf370292-bib-0054] Tortorello, M. L. 2003. “Indicator Organisms for Safety and Quality—Uses and Methods for Detection: Minireview.” Journal of AOAC International 86, no. 6: 1208–1217. 14979704

[crf370292-bib-0055] U.S. Centers for Disease Control and Prevention . 2016. “PulseNet Methods and Protocols.” Accessed July 30. https://www.cdc.gov/pulsenet/pathogens/protocols.html.

[crf370292-bib-0056] U.S. Centers for Disease Control and Prevention . 2022a. “ *Listeria* Outbreak Linked to Enoki Mushrooms—November 2022.” Accessed January 22. https://www.cdc.gov/listeria/outbreaks/enoki‐mushrooms‐11‐22.html.

[crf370292-bib-0057] U.S. Centers for Disease Control and Prevention . 2022b. “ *Listeria* Outbreak Linked to Packaged Salads Produced by Fresh Express.” Accessed November 20. https://www.cdc.gov/listeria/outbreaks/packaged‐salad‐12‐21‐b/index.html.

[crf370292-bib-0058] U.S. Centers for Disease Control and Prevention . 2023a. “CDC *Listeria* Initiative Case Report Form.” Accessed November 21. https://www.cdc.gov/listeria/pdf/listeria‐initiative‐case‐report‐form‐p.pdf.

[crf370292-bib-0059] U.S. Centers for Disease Control and Prevention . 2023b. “ *Listeria* Outbreak Linked to Enoki Mushrooms.” Accessed November 16. https://www.cdc.gov/listeria/outbreaks/enoki‐11‐22/index.html.

[crf370292-bib-0060] U.S. Centers for Disease Control and Prevention . 2024. “About The *Listeria* Initiative.” Accessed November 4. https://www.cdc.gov/listeria/php/surveillance/listeria‐initiative.html#:~:text=The%20Listeria%20Initiative%20is%20a,information%20to%20the%20Listeria%20Initiative.

[crf370292-bib-0061] U.S. Department of Agriculture Economic Research Service . 2017. “Americans Consume Nearly 3 Pounds of Fresh Mushrooms per Year.” Accessed November 20. https://www.ers.usda.gov/data‐products/chart‐gallery/gallery/chart‐detail/?chartId=85803.

[crf370292-bib-0062] U.S. Department of Agriculture Economic Research Service . 2023. “Factors Affecting U.S. Mushroom Consumption.” Accessed November 20. https://www.ers.usda.gov/webdocs/outlooks/39489/30836_vgs29501_002.pdf?v=5962.

[crf370292-bib-0063] U.S. Department of Agriculture National Agricultural Statistics Service . 2022. “Mushrooms.” Accessed November 20. https://www.nass.usda.gov/Publications/Todays_Reports/reports/mush0822.pdf.

[crf370292-bib-0064] U.S. Food and Drug Administration . 2019a. “FDA Strategy for the Safety of Imported Food”. Accessed June 21. https://www.fda.gov/media/120585/download.

[crf370292-bib-0065] U.S. Food and Drug Administration . 2019b. “Import Alerts.” Accessed December 2. https://www.fda.gov/industry/actions‐enforcement/import‐alerts.

[crf370292-bib-0066] U.S. Food and Drug Administration . 2020. “FSMA Final Rule on Produce Safety: Standards for the Growing, Harvesting, Packing, and Holding of Produce for Human Consumption.” Accessed May 22. https://www.fda.gov/food/food‐safety‐modernization‐act‐fsma/fsma‐final‐rule‐produce‐safety.

[crf370292-bib-0067] U.S. Food and Drug Administration . 2021. “Import Alert 99‐23.” Accessed May 19. https://www.accessdata.fda.gov/cms_ia/importalert_266.html.

[crf370292-bib-0068] U.S. Food and Drug Administration . 2022. “Summary of FDA's Strategy to Help Prevent Listeriosis and Salmonellosis Outbreaks Associated with Imported Enoki and Imported Wood Ear Mushrooms.” Accessed September 29. https://www.fda.gov/food/new‐era‐smarter‐food‐safety/summary‐fdas‐strategy‐help‐prevent‐listeriosis‐and‐salmonellosis‐outbreaks‐associated‐imported‐enoki?utm_medium=email&utm_source=govdelivery.

[crf370292-bib-0069] U.S. Food and Drug Administration . 2023a. “FDA Advises Restaurants and Retailers Not to Serve or Sell and Consumers Not to Eat Product Labeled as Sun Hong Foods, Inc. Enoki Mushrooms Sourced from China Due to Possible *Listeria* Contamination.” Accessed December 29. https://www.fda.gov/food/alerts‐advisories‐safety‐information/fda‐advises‐restaurants‐and‐retailers‐not‐serve‐or‐sell‐and‐consumers‐not‐eat‐product‐labeled‐sun.

[crf370292-bib-0070] U.S. Food and Drug Administration . 2023b. “Import Alert 25‐21.” Accessed December 29. https://www.accessdata.fda.gov/cms_ia/importalert_1177.html.

[crf370292-bib-0071] U.S. Food and Drug Administration . 2023c. “Laboratory Flexible Funding Model Cooperative Agreement Program.” Accessed June 29. https://www.fda.gov/federal‐state‐local‐tribal‐and‐territorial‐officials/grants‐and‐cooperative‐agreements/laboratory‐flexible‐funding‐model‐cooperative‐agreement‐program#What%20is%20the%20Lab%20FFM?.

[crf370292-bib-0072] U.S. Food and Drug Administration . 2023d. “Outbreak Investigation of *Listeria monocytogenes*: Enoki Mushrooms (November 2022).” Accessed November 20. https://www.fda.gov/food/outbreaks‐foodborne‐illness/outbreak‐investigation‐listeria‐monocytogenes‐enoki‐mushrooms‐november‐2022.

[crf370292-bib-0073] U.S. Food and Drug Administration . 2024a. “Enoki King Mushroom Farm Recalls Enoki Because of Possible Health Risk.” Accessed November 29. https://www.fda.gov/safety/recalls‐market‐withdrawals‐safety‐alerts/enoki‐king‐mushroom‐farm‐recalls‐enoki‐because‐possible‐health‐risk.

[crf370292-bib-0074] U.S. Food and Drug Administration . 2024b. “Prevention Strategies to Enhance Food Safety.” Accessed May 19. https://www.fda.gov/food/outbreaks‐foodborne‐illness/post‐outbreak‐response‐and‐prevention‐strategies‐enhance‐food‐safety‐updated‐january‐17‐2025.

[crf370292-bib-0075] U.S. Food and Drug Administration . 2025. “Post‐Outbreak Response and Prevention Strategies to Enhance Food Safety (Updated January 17, 2025).” Accessed June 2. https://www.fda.gov/food/outbreaks‐foodborne‐illness/post‐outbreak‐response‐and‐prevention‐strategies‐enhance‐food‐safety‐updated‐january‐17‐2025.

[crf370292-bib-0076] United Kingdom Food Standards Agency . 2023. “Advice on *Listeria monocytogenes* in Imported Enoki Mushrooms.” Accessed November 21. https://www.food.gov.uk/news‐alerts/news/advice‐on‐listeria‐monocytogenes‐in‐imported‐enoki‐mushrooms#:~:text=the%20product%20safely.‐,The%20Food%20Standards%20Agency%20(%20FSA%20)%20and%20Food%20Standards%20Scotland%20(,prepare%20them%20and%20wash%20hands.

[crf370292-bib-0077] Wang, P. M. , X. B. Liu , Y. C. Dai , E. Horak , K. Steffen , and Z. L. Yang . 2018. “Phylogeny and Species Delimitation of *Flammulina*: Taxonomic Status of Winter Mushroom in East Asia and a New European Species Identified Using an Integrated Approach.” Mycological Progress 17: 1013–1030.

[crf370292-bib-0078] Wellman, A. , M. C. Bazaco , T. Blessington , et al. 2023. “An Overview of Foodborne Sample‐Initiated Retrospective Outbreak Investigations and Interagency Collaboration in the United States.” Journal of Food Protection 86, no. 6: 100089. 10.1016/j.jfp.2023.100089. 37024093

[crf370292-bib-0079] World Integrated Trade Solution . 2022. “Vegetables; Mushrooms, Fresh or Chilled Imports by Country in 2022.” Accessed November 20. https://wits.worldbank.org/trade/comtrade/en/country/ALL/year/2022/tradeflow/Imports/partner/WLD/product/070951.

[crf370292-bib-0080] Yoon, J.‐H. , H. Chu , D.‐Y. Jeong , et al. 2020. “Decontamination of *Listeria monocytogenes* in Enoki Mushrooms Using a 405‐nm Light‐Emitting Diode Illumination Combined With Organic Acid Dipping.” LWT 133: 110048. 10.1016/j.lwt.2020.110048. https://www.sciencedirect.com/science/article/pii/S0023643820310379.

